# Expression Profile of Circulating MicroRNAs in Dogs With Cardiac Hypertrophy: A Pilot Study

**DOI:** 10.3389/fvets.2021.652224

**Published:** 2021-04-09

**Authors:** Woong-Bin Ro, Min-Hee Kang, Doo-Won Song, Sung-Hun Lee, Hee-Myung Park

**Affiliations:** ^1^Department of Veterinary Internal Medicine, College of Veterinary Medicine, Konkuk University, Seoul, South Korea; ^2^Department of Cancer Genome Research, Cancer Research Institute, Clinomics Inc., Seoul, South Korea

**Keywords:** microRNA, dog, cardiac hypertrophy, myxomatous mitral valve degeneration, pulmonic stenosis, serum, novel biomarker, therapeutic target

## Abstract

This study aimed to identify the expression profile of circulating microRNAs in dogs with eccentric or concentric cardiac hypertrophy. A total of 291 microRNAs in serum samples of five dogs with myxomatous mitral valve degeneration (MMVD) and five dogs with pulmonic stenosis (PS) were compared with those of five healthy dogs using microarray analysis. Results of microarray analysis revealed up-regulation of cfa-miR-130b [fold change (FC) = 2.13, *p* = 0.014), down-regulation of cfa-miR-375 (FC = 1.51, *p* = 0.014), cfa-miR-425 (FC = 2.56, *p* = 0.045), cfa-miR-30d (FC = 3.02, *p* = 0.047), cfa-miR-151 (FC = 1.89, *p* = 0.023), cfa-miR-19b (FC = 3.01, *p* = 0.008), and cfa-let-7g (FC = 2.53, *p* = 0.015) in MMVD group which showed eccentric cardiac hypertrophy, up-regulation of cfa-miR-346 (FC = 2.74, *p* = 0.032), down-regulation of cfa-miR-505 (FC = 1.56, *p* = 0.016) in PS group which showed concentric cardiac hypertrophy, and down-regulation of cfa-miR-30c (FC = 3.45, *p* = 0.013 in MMVD group; FC = 3.31, *p* = 0.014 in PS group) and cfa-let-7b (FC = 11.42, *p* = 0.049 in MMVD group; FC = 5.88, *p* = 0.01 in PS group) in both MMVD and PS groups. In addition, the unsupervised hierarchical clustering of differentially expressed microRNAs in each group resulted in complete separation of healthy dogs from dogs with heart diseases. Therefore, eleven microRNAs among 291 microRNAs were identified as differentially expressed circulating microRNAs related to MMVD or PS in dogs. This pilot study demonstrates that the microRNAs identified in this study could be possible candidates for novel biomarker or therapeutic target related to cardiac hypertrophy in dogs.

## Introduction

Cardiac hypertrophy is an enlargement of the heart, which occurs in response to increased cardiac workload caused by various heart diseases (1). There are two types of cardiac hypertrophy such as eccentric and concentric cardiac hypertrophy. The eccentric cardiac hypertrophy is caused by volume overload diseases such as myxomatous mitral valve degeneration (MMVD), patent ductus arteriosus, and dilated cardiomyopathy in dogs, and results in increased myocyte length and dilated chambers with variable relative wall thickness (RWT) (2). The concentric cardiac hypertrophy is caused by pressure overload diseases such as pulmonic stenosis (PS) and aortic stenosis in dogs, and results in increased myocyte width and RWT with normal to decreased chamber volume (2).

MicroRNAs (miRNAs) are single-stranded, small non-coding RNAs containing 18–22 nucleotides that pair with specific target mRNAs to regulate and inhibit gene expression by degradation or translation inhibition (3). Previous studies have reported that miRNA play crucial roles in heart development and pathophysiology of cardiovascular diseases (4). In addition, circulating microRNAs are increasingly recognized as promising biomarkers for heart diseases because of their stability in peripheral blood (5). In humans, significant dysregulations of circulating microRNAs in cardiovascular diseases have been reported (5), and the role of microRNAs in cardiac hypertrophy has been identified (6–8). Moreover, the microRNAs related to cardiac hypertrophy are increasingly considered as potential therapeutic targets for various heart diseases (9).

Previous studies on circulating microRNAs in dogs with naturally occurring heart diseases are limited in number, and the studies have been conducted only in dogs with eccentric cardiac hypertrophy, such as MMVD and dilated cardiomyopathy (10–14). Therefore, the expression of microRNAs specific for concentric cardiac hypertrophy such as PS has not been studied yet in dogs. In this study, we aimed to demonstrate expression profiles of circulating microRNAs in dogs with eccentric or concentric cardiac hypertrophy, and investigate whether there is a difference in microRNA expression according to the type of cardiac hypertrophy. Dogs with MMVD or PS were included in this study, which are the two representative diseases of eccentric or concentric cardiac hypertrophy in dogs, respectively. We hypothesized that circulating microRNAs would be differentially expressed according to the type of cardiac hypertrophy or type of heart disease.

## Materials and Methods

### Serum Samples

Stored serum samples of five dogs with MMVD and with PS each were retrospectively retrieved and used in this study.

The inclusion criteria for MMVD group were dogs with MMVD of American College of Veterinary Internal Medicine (ACVIM) stage C or D, which were diagnosed and staged according to the guidelines of the ACVIM Consensus Statement for canine mitral valve disease, as previously described (15). According to the ACVIM guideline (15), stage C referred to dogs with past or current clinical signs by MMVD, and stage D indicated end-stage MMVD dogs, in which the clinical signs of heart failure were refractory to standard treatment used in stage C.

The inclusion criteria for PS group were dogs with PS of moderate or severe stenosis, which were diagnosed and staged according to the echocardiographic criteria for PS in dogs (16). According to the criteria (16), moderate stenosis referred to right ventricular outflow tract (RVOT) peak pressure gradient from 50 to 80 mmHg, and severe stenosis referred to RVOT peak pressure gradient above 80 mmHg.

Although the stages of MMVD and PS cannot be compared directly, progressed stages of the two diseases (stage C or D in MMVD, moderate or severe stenosis in PS) were included in this study in order to match the severity of the two diseases, as well as to ensure the eccentric and concentric cardiac hypertrophy in MMVD and PS groups, respectively.

The exclusion criteria for both MMVD and PS groups were dogs with any diseases other than MMVD or PS that could possibly affect circulating microRNA expression or cardiac hypertrophy, such as concurrent heart diseases, neoplasia, hypothyroidism, hyperadrenocorticism, diabetes mellitus, and systemic hypertension.

Five stored serum samples from healthy beagle dogs were used and they were retrieved from the remaining of a previous study (17). The previous study was approved by KBNP Institutional Animal Care and Use Committee (R0006046). The serum samples were not affected by the previous study because the blood was collected before initiation of the previous study, and were stored for 3 months before analysis. All healthy dogs in the previous study were examined to be healthy by physical examination, and there were no remarkable findings in complete blood count, serum chemistry, and urinalysis.

Blood was collected from jugular vein into 5-ml serum separating tubes (BD Vacutainer® SST™ Tube, USA). The tube was gently inverted about 5 times and was allowed to stand for 20–30 min at 4°C before centrifugation at 3,000 rpm for 15 min. The serum was aliquoted into cryovials (SPL Life Sciences, Korea) and stored at−70°C until use.

A total of 15 clinical data including breed, age, sex, heart rate, blood pressure, and results of physical examination were retrieved and evaluated from medical records from December 2014 to September 2018.

### Echocardiographic Assessments

Echocardiographic data were acquired from the medical records. The EPIQ 7 ultrasound system (Philips Medical Systems, Andover, MA, USA) was utilized. A short-axis images of left ventricle (LV) were obtained at the left atrium and papillary muscle levels from the right-parasternal view. The values of LV measurements were normalized to the body weight according to the previous study (18). The fractional shortening (FS), ejection fraction (EF), end-diastolic volume index (EDVI), end-systolic volume index (ESVI), ratio of left atrial to aortic root diameter (LA/AO), normalized value of end-diastolic LV internal dimension (LVIDDN), normalized value of end-systolic LV internal dimension (LVIDSN), normalized value of end-diastolic LV free wall thickness (LVPWDN), normalized value of end-systolic LV free wall thickness (LVPWSN), normalized value of end-diastolic interventricular septal thickness (IVSDN), normalized value of end-systolic interventricular septal thickness (IVSSN), and RWT were analyzed for evaluation of cardiac function, hypertrophy, and remodeling of dogs included in this study. The RWT was calculated by following formula: RWT = (end-diastolic interventricular septal thickness + end-diastolic LV free wall thickness)/end-diastolic LV internal dimension (19).

### RNA Preparation

RNA was extracted from serum using the miRNeasy Serum/Plasma Kit (Qiagen, Hilden, Germany) according to the manufacturer's protocol. RNA integrity and quantity were evaluated with nanodrop 1000 Spectrophotometer (Thermo Fisher Scientific, Waltham, MA, USA), Quant-IT microRNA assay kit (Thermo Fisher Scientific) by QuantusTM Fluorometer (Promega, Madison, WI, USA), and Agilent 2100 Bioanalyzer (Agilent Technologies, Palo Alto, USA).

### Gene Microarray Hybridization

The RNA was labeled using the FlashTag^TM^ Biotin RNA Labeling Kit (Affymetrix, Santa Clara, CA, USA) and hybridized to GeneChip miRNA 4.0 microarrays (Affymetrix).

A hybridization mixture was added to samples with controls including control oligo B2, 20 X hybridization controls (bioB, bioC, bioD, cre), 27.5% Formamide, DMSO, 2 X hybridization buffer and water. 110.5 μl of this mixture was injected into GeneChip miRNA 4.0 arrays containing 291 canine probes annotated in miRBase Release 20, and placed in the Affymetrix GeneChip Hybridization Oven 640 at 48 °C and 60 rpm for 16 h overnight. The information on 291 microRNAs investigated in this study are shown in Supplementary Table 1. To amplify singal intensities, stain cocktails (stain cocktail 1 and 2) were added. Arrays were stained and washed using the Affymetrix GeneChip Fluidics Station 450 according to the FS450_0002 fluidics protocol.

### Microarray Scanning and Data Processing

All arrays were scanned using the Affymetrix GeneChip Scanner 3000 (Affymetrix) and raw analysis performed with Transcriptome Analysis ConsoleTM (TAC) software (Affymetrix). The raw data images generated from the scanner were processed into CEL files, that contained measured intensities for each probe on the array. The fold change of > 1.5 (up or down regulation) and *p*-value of < 0.05 was used as a cut-off to screen wide range of differentially expressed microRNAs.

### Statistical Analysis

The clinical data of dogs included in this study were presented as the mean ± standard deviation. The different expressions of microRNAs were compared between healthy vs. MMVD groups and healthy vs. PS groups, using Mann-Whitney test. The values of clinical and echocardiographic data for three groups were compared using Kruskal-Wallis test and *post-hoc* test. Statistical analysis was performed using the SPSS 25.0 software (SPSS Inc., Chicago, IL, USA). A *p*-value was considered statistically significant at the less of 0.05. Unsupervised hierarchical clustering of differentially expressed microRNAs was performed by Multi Experiment Viewer (MeV) software version 4.9.0, using average link hierarchical clustering and Euclidian distance measure.

## Results

**Figure 1:**
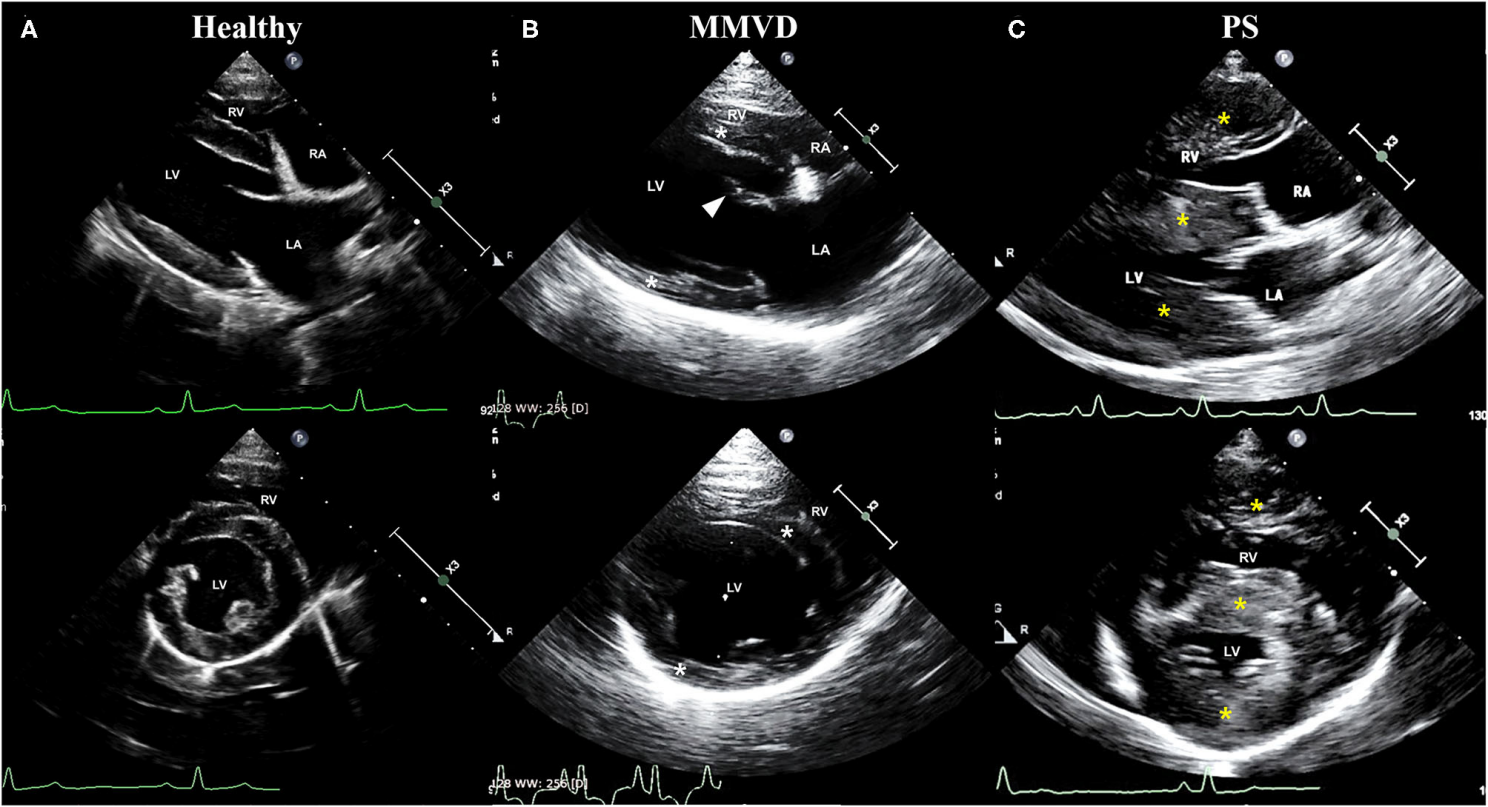
Representative echocardiographic images of dogs included in **(A)** healthy, **(B)** MMVD, and **(C)** PS groups. Upper images are right parasternal 4-chamber view, and lower images are right parasternal short-axis view. All images were taken during the diastole. **(B)** Note the enlargement of chambers (LV and LA), eccentric hypertrophy of ventricular walls (white asterisk), and thickened mitral valve (arrowhead) in a dog with MMVD. **(C)** Also, note the concentric hypertrophy of ventricular walls (yellow asterisk) in a dog with PS. LA, left atrium; LV, left ventricle; MMVD, myxomatous mitral valve degeneration; PS, pulmonic stenosis; RA, right atrium; RV, right ventricle.

The clinical characteristics of dogs included in this study are shown in Table 1. The breed of dogs included in this study were five Beagle in healthy group, four Maltese and one Shih Tzu in MMVD group, and two Pomeranian, one French Bulldog, one Mixed-breed, and one Poodle in PS group. The mean age of dogs with MMVD was significantly higher than that of other groups (*p* = 0.007), and the mean diastolic blood pressure of dogs with PS was significantly higher when compared with healthy dogs (*p* = 0.039). In echocardiographic analysis, the FS and EF were significantly increased in both MMVD and PS group compared with healthy group (*p* = 0.006 and *p* = 0.006, respectively). The EDVI of PS group was significantly lower than that of healthy group (*p* = 0.039), and the ESVI was significantly decreased in both MMVD and PS group compared with healthy group (*p* = 0.004). The LA/AO of MMVD group was significantly higher than that of other groups (*p* = 0.002). The LVIDDN was significantly different between the three groups (*p* = 0.002), the highest in MMVD group and the lowest in PS group. The LVIDSN of PS group was significantly lower than that of other groups (*p* = 0.007). The IVSDN and IVSSN of PS group were significantly higher than those of healthy group (*p* = 0.030 and *p* = 0.041, respectively), and the RWT of dogs with PS was significantly higher than that of other groups (*p* = 0.007). Of the five dogs with MMVD, two dogs were stage C and three dogs were stage D based on the ACVIM staging system. All dogs with PS presented severe stenosis in valvular form. The representative echocardiographic images of dogs included in this study are shown in Figure 1.

**Table 1:** Clinical characteristics of dogs included in this study.

**Variables**	**Healthy (*n* = 5)**	**MMVD (*n* = 5)**	**PS (*n* = 5)**	**Reference value**
Age, year	2.54 ± 0.54	12.83 ± 2.01^a^	2.17 ± 1.99^b^	
Male/female, *n*	5/0	3/2	3/2	
Heart rate, bpm	126 ± 12	142 ± 17	129 ± 13	70–160 (20)
Systolic BP, mmHg	127 ± 9	134 ± 12	140 ± 10	90–140 (21)
Diastolic BP, mmHg	79 ± 4	91 ± 12	100 ± 12^a^	50–80 (21)
**Echocardiography**				
FS, %	37.27 ± 1.63	60.82 ± 8.92^a^	51.04 ± 8.60^a^	25–50 (22)
EF, %	68.61 ± 2.09	90.38 ± 6.17^a^	84.20 ± 7.13^a^	59–83 (23)
EDVI	81.74 ± 5.15	95.83 ± 56.69	26.89 ± 11.12^a^	44–117 (23)
ESVI	25.68 ± 2.65	9.80 ± 4.15^a^	4.48 ± 3.10^a^	9–38 (23)
LA/AO	1.16 ± 0.08	2.44 ± 0.69^a^	1.36 ± 0.11^a^^,^ ^b^	0.8–1.3 (18)
LVIDDN	1.61 ± 0.05	1.91 ± 0.12^a^	1.02 ± 0.17^a^^,^ ^b^	1.35–1.73 (18)
LVIDSN	0.98 ± 0.07	0.73 ± 0.18	0.48 ± 0.13^a^^,^ ^b^	0.79–1.14 (18)
LVPWDN	0.44 ± 0.06	0.41 ± 0.06	0.58 ± 0.23	0.33–0.53 (18)
LVPWSN	0.66 ± 0.08	0.81 ± 0.16	0.83 ± 0.24	0.53–0.78 (18)
IVSDN	0.46 ± 0.08	0.45 ± 0.13	0.63 ± 0.06^a^	0.33–0.52 (18)
IVSSN	0.66 ± 0.03	0.85 ± 0.17	0.77 ± 0.07^a^	0.48–0.71 (18)
RWT	0.49 ± 0.07	0.43 ± 0.10	1.13 ± 0.44^a^^,^ ^b^	–

*BP, blood pressure; EDVI, end-diastolic volume index; EF, ejection fraction; ESVI, end-systolic volume index; FS, fractional shortening; IVSDN, normalized value of end-diastolic interventricular septal thickness; IVSSN, normalized value of end-systolic interventricular septal thickness; LA/AO, ratio of left atrial to aortic root diameter; LVIDDN, normalized value of end-diastolic left ventricular internal dimension; LVIDSN, normalized value of end-systolic left ventricular internal dimension; LVPWDN, normalized value of end-diastolic left ventricular free wall thickness; LVPWSN, normalized value of end-systolic left ventricular free wall thickness; MMVD, myxomatous mitral valve degeneration; PS, pulmonic stenosis; RWT, relative wall thickness*.

*Continuous variables were expressed as mean ± standard deviation*.

a *p < 0.05 compared with Healthy group*,

b *p < 0.05 compared with MMVD group*.

**Figure 2:**
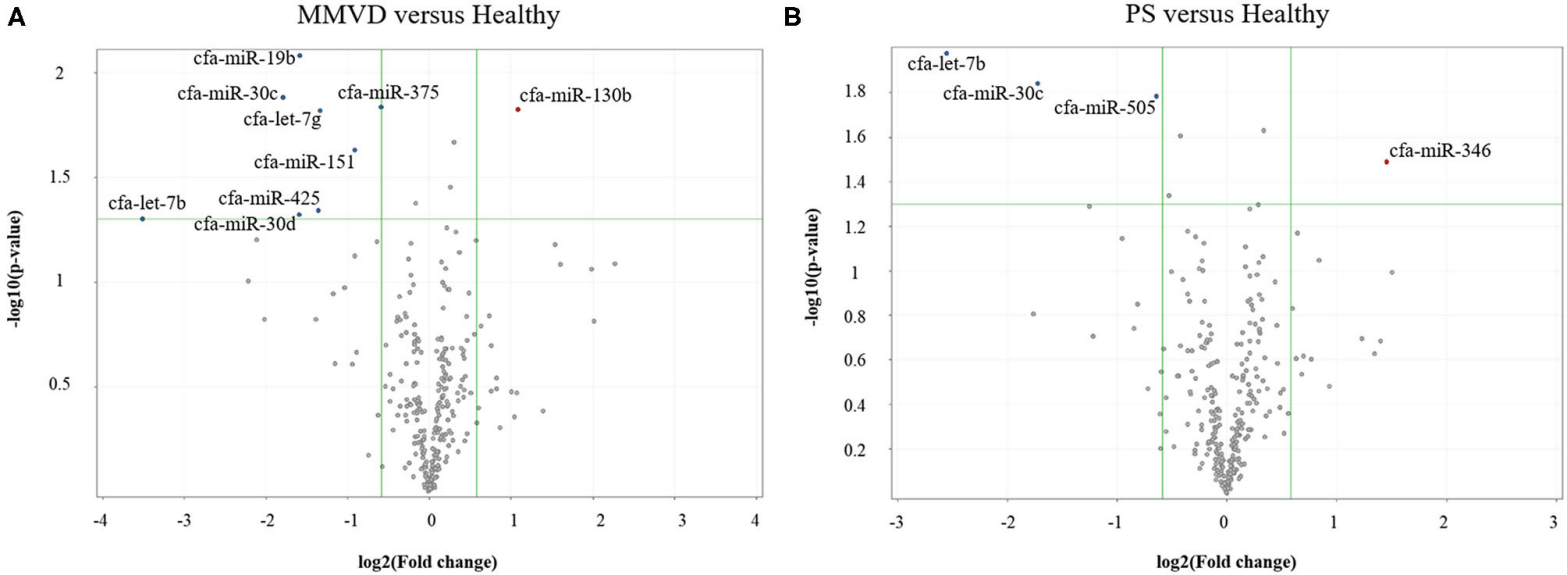
Volcano plots of circulating miRNA in dogs with **(A)** MMVD or **(B)** PS, compared with healthy dogs. The volcano plot shows the fold change and *p*-value between the dogs with heart diseases and healthy dogs for 291 miRNAs. The vertical lines correspond to a 1.5-fold differences, and the horizontal line displayed a *p*-value of 0.05. Therefore, the blue and red points in the plot represent the miRNAs with statistically significant differential expression in dogs with MMVD or PS (A or B, respectively) compared with healthy dogs. miRNA, microRNA; MMVD, myxomatous mitral valve degeneration; PS, pulmonic stenosis.

**Figure 3:**
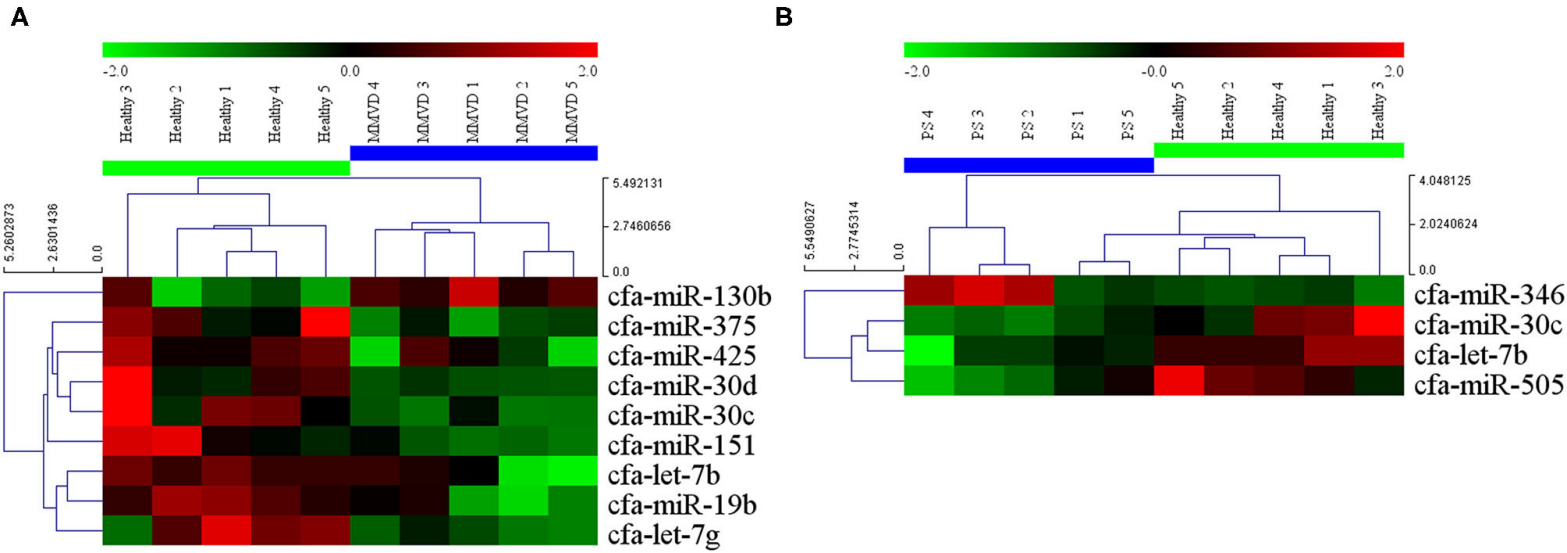
Heat map of significantly altered miRNA microarray expression data from serum samples of dogs with **(A)** MMVD and **(B)** PS compared with healthy dogs. Sample species are shown at the top and the miRNA species are shown on the right. Unsupervised hierarchical clustering of expression levels of nine and four (**A** and **B**, respectively) differentially expressed microRNAs using the Euclidian distance measure. The hierarchical clustering completely separated healthy dogs from dogs with heart diseases, showing distinct microRNA patterns among sample types. Red and green colors indicate relatively high and low expressions of microRNA, respectively. miRNA, microRNA; MMVD, myxomatous mitral valve degeneration; PS, pulmonic stenosis.

The circulating microRNA expression levels of dogs with MMVD or PS, compared with those of the healthy dogs are illustrated in the volcano plots in Figure 2. Using two independent parameters, a fold change cut-off of > 1.5 (up or down regulation) and *p*-value of <0.05, one microRNA (cfa-miR-130b) was significantly up-regulated and eight microRNAs (cfa-miR-375, cfa-miR-425, cfa-miR-30d, cfa-miR-30c, cfa-miR-151, cfa-let-7b, cfa-miR-19b, cfa-let-7g) were significantly down-regulated in dogs with MMVD when compared to healthy dogs (Figure 2A, Supplementary Table 2). In addition, one microRNA (cfa-miR-346) was significantly up-regulated and three microRNAs (cfa-miR-30c, cfa-let-7b, cfa-miR-505) were significantly down-regulated in dogs with PS when compared to healthy dogs (Figure 2B, Supplementary Table 2). The unsupervised hierarchical clustering of differentially expressed microRNAs resulted in complete separation of healthy dogs from dogs with heart diseases, exhibiting distinct microRNA expression patterns according to the sample types (Figure 3). Therefore, eleven microRNAs among 291 microRNAs were identified as differentially expressed circulating microRNAs related to MMVD or PS in dogs.

## Discussion

Myxomatous mitral valve degeneration is the most common heart disease in dogs, and considered as excellent disease model for studying mechanisms that cause eccentric cardiac hypertrophy (24, 25). Pulmonic stenosis is the most common among heart diseases that cause concentric cardiac hypertrophy in dogs, and is characterized by right ventricular (RV) concentric hypertrophy, often RV dilation due to tricuspid regurgitation, and LV pseudo-hypertrophy secondary to decreased LV filling (26–28).

In this study, the distinct characteristics of eccentric and concentric cardiac hypertrophy of dogs with MMVD and PS, respectively, were confirmed by the significant differences in echocardiographic indices between the three groups (Table 1). In addition, similarly matched severity of MMVD and PS in this study ensured that substantial and similar degree of eccentric and concentric cardiac hypertrophy occurred in MMVD and PS groups, respectively. The remarkable increase of LA/AO and LVIDDN in MMVD group indicated prominent cardiac remodeling by volume overload. The RV concentric hypertrophy in PS group was confirmed by septal hypertrophy (increased IVSDN and IVSSN), which is known to occur secondary to increased RV afterload in dogs with PS (29). This septal hypertrophy in PS group may have been also partially contributed by LV pseudo-hypertrophy, which is characterized by decreased LV filling (decreased EDVI, ESVI, LA/AO, LVIDDN, LVIDSN) and consequent pseudo-increased LV wall thickness (IVSDN, IVSSN and RWT). The increase of mean systolic and diastolic blood pressure in PS group may have been due to increased systemic vascular resistance, as previously described in dogs with pulmonary artery occlusion (30) (Table 1). However, there was no statistical significance in systolic blood pressure, which is thought to be due to small sample size.

Of the eleven significantly dysregulated microRNAs in this study, miR-30c and let-7b were commonly dysregulated in both MMVD and PS groups. Therefore, these two microRNAs are thought to be involved in common pathway related to both eccentric and concentric cardiac hypertrophy in dogs.

In a previous study (31), miR-30c was down-regulated in pathological concentric cardiac hypertrophy, and the direct target gene of miR-30c was mRNA of connective tissue growth factor (CTGF), a key pro-fibrotic protein. In addition, down-regulation of circulating miR-30c was also observed in both eccentric and concentric cardiac hypertrophy, in dogs with MMVD and in heart failure patients with LV concentric hypertrophy, respectively (11, 32). Taken together, these results indicate that the down-regulation of miR-30c is associated with both eccentric and concentric cardiac hypertrophy, and the decrease of miR-30c may cause accumulation of CTGF, contributing to disease progression by pro-fibrotic effect. These results are consistent with the results of the present study, which showed down-regulation of miR-30c in both eccentric and concentric cardiac hypertrophy groups.

According to previous studies (33, 34), let-7b is known to be up-regulated by thioredoxin1 (Trx1), which is increased in response to various stress to heart including ischemia, heart failure, and pressure overload. These heart conditions such as ischemia and heart failure are common pathologic states that can be resulted from both eccentric and concentric cardiac hypertrophy, which explains the dysregulation of let-7b in both MMVD and PS groups in this study. In addition, it is reported that the let-7b up-regulated by Trx1 inhibits angiotensin-II induced cardiac hypertrophy by regulation of target, cyclin D2 (33). This anti-hypertrophic property of let-7b implicates its potential as promising therapeutic target in dogs with cardiac hypertrophy.

Meanwhile, let-7b was up-regulated in MMVD dogs in a previous study (12), whereas down-regulation was identified in this study. Similarly, discrepant expression of miR-30c was reported in a previous study in rats, in which miR-30c was down-regulated in heart failure but also showed up-regulation in response to therapeutic treatment (35). This opposite direction of regulation related to therapeutic treatment was also observed in previous studies on let-7b, which showed up-regulation in heart failure and down-regulation in response to treatment (35, 36). Based on these results, miR-30c and let-7b are thought to be associated with not only cardiac hypertrophy but also recovery of cardiac condition by treatment, and the expression of those microRNAs in heart diseases may be different depending on the stage of the heart disease or treatment status. For example, the let-7b may be up-regulated when the anti-hypertrophic compensatory mechanism is still working, however down-regulation of let-7b would be observed in non-compensated stage of heart disease. However, the reason of down-regulation of let-7b in response to treatment is required to be clarified in further studies. Meanwhile, the previous study in MMVD dogs reported that the expression level of let-7b showed tendency to be positively correlated to severity of MMVD (12). Although several mechanisms of gene regulation by miR-30c and let-7b in heart diseases have been identified in previous studies (31, 37–39), further studies on specific roles of these microRNAs in dogs with various cardiac states are required.

In this study, seven microRNAs (miR-130b, miR-375, miR-425, miR-30d, miR-151, miR-19b, let-7g) were significantly dysregulated in MMVD group. According to the previous studies (40, 41), miR-130b was reported to inhibit CYLD, which mediates lesion formation of vessels and cardiomyocytes, leading to cardiac dysfunction. The recovery of heart condition by alteration of microRNA was also reported in previous studies on miR-375 (42, 43), in which the inhibition of miR-375 attenuated inflammation and LV dysfunction, and also enhanced myocardial repair and function after myocardial infarction. The miR-425 has been reported to have anti-fibrotic effect on heart by inhibiting TGFβ1 (44). The expression of miR-30d, which is selectively enriched in cardiomyocytes, is induced by hypoxic stress and inhibits MAP4K4 to reduce cardiac apoptosis (45). The miR-151 targets PLM and is known to prevent ischemia-induced arrhythmias (46). The miR-19b also has cardio-protective property to regenerate cardiomyocyte in response to myocardial infarction (47). Lastly, let-7g inhibits cell adhesion and inflammation, while enhancing angiogenesis by targeting THBS1, which is involved in TGF-β pathway (48).

All these microRNAs dysregulated in MMVD group were reported to be dysregulated in previous studies on heart diseases in humans and rats (32, 35, 36, 42–44, 49–51), and four microRNAs among them (miR-130b, miR-425, miR-19b, let-7g) were identified to be dysregulated in previous studies in dogs with MMVD (11, 13). In the previous studies in dogs (11, 13), these four microRNAs showed down-regulation, which is consistent with the results of the present study except for miR-130b.

Contrary to the findings from previous studies in dogs (11, 13), up-regulation of miR-130b was observed in MMVD group in this study, and also in previous studies in rats and mice with heart diseases (49, 51). This discrepant expressions of miR-130b may be explained by the same reason as miR-30c and let-7b mentioned above. In the previous study in rats with induced cardiac hypertrophy (36), miR-130b was initially down-regulated in cardiac hypertrophy, but showed up-regulation in reverse-remodeling process in which the heart condition was restored. Therefore, similar to miR-30c and let-7b, miR-130b may be associated with protection of heart from cardiac hypertrophy and also recovery of cardiac condition by reverse-remodeling. In a previous human study (52), the miR-130b was down-regulated in the plasma of obese heart failure patients, and the biological pathways of predicted target genes of miR-130b were associated with prevention of cardiac hypertrophy, cardiomyocyte differentiation, and cardiac remodeling. This result corresponds to the results of the present and previous studies (11, 36, 49, 51). As mentioned above, miR-130b was reported to be protective for cardiac lesion and heart dysfunction (40, 41). Therefore, the up-regulation of miR-130b in MMVD group in this study may indicate that the miR-130b related protective pathway was activated in order to attenuate the progression of disease. However, this is difficult to be confirmed from the results of this study because serial evaluation of heart condition was not conducted. In addition, pathway causing miR-130b to be dysregulated is not identified clearly in dogs with heart diseases, therefore these speculations are needed to be verified in future studies.

Meanwhile, six microRNAs that were dysregulated only in MMVD group in this study (miR-130b, miR-375, miR-30d, miR-151, miR-19b, let-7g) were dysregulated also in concentric cardiac hypertrophy in previous murine studies (35, 36). In addition, as mentioned above, miR-425 was associated with cardiac fibrosis, which is a common pathologic change that can be resulted from both eccentric and concentric cardiac hypertrophy (44, 53). Therefore, although these seven microRNAs were significantly dysregulated only in MMVD group in this study, they are considered to be microRNAs that can be observed in both eccentric and concentric cardiac hypertrophy. The possible reasons why these microRNAs were not identified in the PS group in this study may include small number of samples and variable expression of microRNAs depending on the various factors, such as disease states as well as disease type.

The miR-346 and miR-505, which showed dysregulations only in the PS group, have not been reported in dogs until now. The target of miR-346 is Bax, which is a regulator of apoptosis, thus miR-346 inhibits apoptosis in myocardial ischemia-reperfusion injury (54). The up-regulation of miR-346 was observed in dogs with PS in this study, which is consistent with the result of previous study in which miR-346 was significantly up-regulated in ischemia reperfusion injured mouse hearts (55). Meanwhile, a previous study reported that miR-346 was up-regulated in ischemic myocardium of rats with myocardial infarction, and silencing of miR-346 significantly inhibited inflammatory response and apoptosis of cardiomyocytes (56). Since hearts with concentric hypertrophy are known to be susceptible to ischemic injury (57), miR-346 may have been predominantly expressed in dogs with PS in this study. The miR-505 was down-regulated in dogs with PS in this study. This microRNA interferes vascular regeneration by targeting FGF18 (58). In a previous study in neonatal mouse heart (59), miR-505-5p was down-regulated in heart tissue of 7-day-old mice compared to heart tissue of 1- and 6-day-old mice, and the target genes of miR-505-5p was associated with myocardial regenerative process. On the other hand, the up-regulation of miR-505-5p was observed in human patients with degenerative aortic stenosis. Since miR-346 and miR-505 were dysregulated only in PS group in this study and not identified in previous studies in dogs with MMVD, these microRNAs can be possibly associated with specific changes of PS or concentric cardiac hypertrophy.

It should be noted that the target genes or proteins, and related pathways of microRNAs described in this study were previously confirmed in other species, rather than dogs. Although most microRNAs and their targets are conserved among animals with different species, the conserved microRNAs do not always show the same expression patterns or levels in different species (60). In addition, multiple microRNAs are related to multiple target genes, thus identification of disease-specific target gene that is actually related to pathologic pathways is challenging (61). Therefore, further identification and validation of target genes in dogs are warranted.

The relationship of circulating microRNA with cardiac tissue microRNA remains largely unknown. However, recent study in humans showed strong positive correlation of circulating microRNA with pericardial microRNAs (62). In addition, several previous studies revealed that cardiac-enriched microRNAs were released to circulation in response to myocardial injury (63, 64). These results have raised potential of circulating microRNAs as novel diagnostic and prognostic biomarker. Nevertheless, there are still many challenges to use microRNAs as useful biomarker or therapeutic targets.

Despite of promising characteristics of circulating microRNAs, identifying the source of circulating microRNAs is very difficult, because many microRNAs have ubiquitous characteristics, and are related to variety of different clinical states (65). Therefore, it is unsure if circulating microRNAs identified in this study are representatives for cardiac microRNA. In addition, low expression of circulating microRNAs makes it more challenging to detect and accurately quantify them (66). Therefore, still large gap remains before clinical application of circulating microRNAs, which requires accumulation of further studies to identify and validate specific characteristics of circulating microRNAs.

This study has several limitations. First, the breed, age, and sex of healthy dogs were not matched with those of dogs with heart diseases, not satisfying the homogeneity of sample groups. In previous human studies (67–70), racial or ethnic difference, age, and sex were reported to be related to microRNA expression. However, association of microRNA expression with breed, age, and sex is not identified in dogs yet, which needs to be clarified in the future. Second, primary cardiac hypertrophy due to genetic cause could not be evaluated, because dogs with congenital cardiomyopathy were not included in this study. Third, the cardiac hypertrophy may not be the only factor associated with expression of circulating microRNAs in this study. Since the cardiac hypertrophy of dogs included in this study was one of various secondary changes to primary heart diseases, the changes in microRNA expression can be associated with not only cardiac hypertrophy but also other conditions such as disease type, myocardial infarction, cardiac fibrosis, and heart failure, as previously reported (71–73). In addition, the different location of cardiac hypertrophy between the two groups (LV hypertrophy in MMVD and RV hypertrophy in PS) should also be considered as another factor that can be associated with different microRNA expression. The factors associated with microRNAs are expected to be clarified by further studies on target genes and specific pathways of each candidate microRNA identified in this study. Lastly, due to small sample size and variable expression of microRNAs in different disease states, there may be unidentified microRNAs in either MMVD or PS groups in this study. Although the design of this study and analysis on the results were conducted according to pre-specified protocol, the results of this study should be verified by more specific study design. Further large-scale follow-up studies are required to validate and evaluate the microRNAs identified in this study as possible biomarkers and therapeutic targets in dogs with cardiac hypertrophy.

This study identified and demonstrated dysregulations of circulating microRNAs in dogs with two different types of cardiac hypertrophy. In addition, the expression profile of circulating microRNAs in dogs with PS was investigated for the first time in this study. This study provides a useful preliminary result for further validation and evaluation of microRNAs as biomarkers or therapeutic targets in dogs with eccentric or concentric cardiac hypertrophy.

## Data Availability Statement

The datasets presented in this study can be found in online repositories. The names of the repository/repositories and accession number(s) can be found below:ArrayExpress database at EMBL-EBI (www.ebi.ac.uk/arrayexpress) under accession number “E-MTAB-10029” (https://www.ebi.ac.uk/arrayexpress/experiments/E-MTAB-10029).

## Ethics Statement

Ethical review and approval was not required for the animal study because stored serum samples were retrospectively used in this study. Informed consent was obtained from the owner for sample collection of client-owned dog. The serum samples of healthy dogs were retrieved from the remaining of a previous study (Ro W-B, Song D-W, Kim KH, Jeong SH, Kang MH. Pharmacokinetics and Pharmacodynamics of Pimobendan-Pentoxifylline Liquid Mixture After Oral Administration in Dogs. J Vet Clin (2019) 36(3):159-65.), which was approved by KBNP Institutional Animal Care and Use Committee (R0006046).

## Author Contributions

W-BR, M-HK, S-HL, and H-MP conceived and designed the study. W-BR and D-WS curated the data and carried out the research. W-BR, M-HK, D-WS and H-MP analyzed the data. W-BR wrote the manuscript. All authors contributed to the article and approved the submitted version.

## Conflict of Interest

S-HL was employed by the company Clinomics Inc. The remaining authors declare that the research was conducted in the absence of any commercial or financial relationships that could be construed as a potential conflict of interest.
