# Erratum: Expression Profile of Circulating MicroRNAs in Dogs With Cardiac Hypertrophy: A Pilot Study

**DOI:** 10.3389/fvets.2021.693891

**Published:** 2021-04-27

**Authors:** Frontiers Production Office

**Affiliations:** Frontiers Media SA, Lausanne, Switzerland

**Keywords:** microRNA, dog, cardiac hypertrophy, myxomatous mitral valve degeneration, pulmonic stenosis, serum, novel biomarker, therapeutic target

Due to a production error, the incorrect supplementary file “Data Sheet 1” was published.

The correct file is published with this erratum, and the original article has been updated.

The publisher apologizes for this mistake. The original article has been updated.

