# Supplementary material for: Expression Profile of Circulating MicroRNAs in Dogs With Cardiac Hypertrophy: A Pilot Study
Source: Front Vet Sci. 2021 Apr 9;8:652224. doi: 10.3389/fvets.2021.652224 (PMC8062772; doi:10.3389/fvets.2021.652224)
Supplement: Supplementary file 1 [file Data_Sheet_1.docx]

Supplementary Material

# Supplementary Table

**Supplementary Table 1.** The sequence and accession number of 291 miRNAs investigated in this study

| **miRNA Name** | **Sequence** | **Sequence Length** | **Accession** |
| --- | --- | --- | --- |
| cfa-miR-448 | UUGCAUAUGUAGGAUGUCCCAU | 22 | MIMAT0001535 |
| cfa-miR-429 | UAAUACUGUCUGGUAAUGCCGU | 22 | MIMAT0001539 |
| cfa-miR-365 | UAAUGCCCCUAAAAAUCCUUAU | 22 | MIMAT0001540 |
| cfa-miR-449 | UGGCAGUGUAUUGUUAGCUGGU | 22 | MIMAT0001544 |
| cfa-miR-450a | UUUUUGCGAUGUGUUCCUAAUA | 22 | MIMAT0001548 |
| cfa-miR-216b | AAAUCUCUGCAGGCAAAUGUGA | 22 | MIMAT0006592 |
| cfa-miR-33a | GUGCAUUGUAGUUGCAUUGC | 20 | MIMAT0006593 |
| cfa-let-7a | UGAGGUAGUAGGUUGUAUAGUU | 22 | MIMAT0006594 |
| cfa-miR-26a | UUCAAGUAAUCCAGGAUAGGCU | 22 | MIMAT0006595 |
| cfa-miR-1835 | UGCACCCUGAGAGCUGGAGCAG | 22 | MIMAT0006596 |
| cfa-miR-32 | UAUUGCACAUUACUAAGUUGCAU | 23 | MIMAT0006597 |
| cfa-miR-204 | UUCCCUUUGUCAUCCUAUGCCU | 22 | MIMAT0006598 |
| cfa-miR-31 | AGGCAAGAUGCUGGCAUAGCUGU | 23 | MIMAT0006599 |
| cfa-miR-101 | UACAGUACUGUGAUAACUGA | 20 | MIMAT0006600 |
| cfa-miR-371 | ACUCAAAAAAUGGCGGCACUUU | 22 | MIMAT0007747 |
| cfa-miR-491 | CUUAUGCAAGAUUCCCUUCUA | 21 | MIMAT0006601 |
| cfa-miR-150 | UCUCCCAACCCUUGUACCAGUG | 22 | MIMAT0006602 |
| cfa-miR-455 | UAUGUGCCUUUGGACUACAUCG | 22 | MIMAT0006603 |
| cfa-miR-30a | UGUAAACAUCCUCGACUGGAAGC | 23 | MIMAT0006604 |
| cfa-miR-30c | UGUAAACAUCCUACACUCUCAGCU | 24 | MIMAT0006605 |
| cfa-miR-206 | UGGAAUGUAAGGAAGUGUGUGG | 22 | MIMAT0006606 |
| cfa-miR-99b | CACCCGUAGAACCGACCUUGCG | 22 | MIMAT0006607 |
| cfa-let-7e | UGAGGUAGGAGGUUGUAUAGUU | 22 | MIMAT0006608 |
| cfa-miR-125a | UCCCUGAGACCCUUUAACCUGU | 22 | MIMAT0006609 |
| cfa-let-7f | UGAGGUAGUAGAUUGUAUAGUU | 22 | MIMAT0006610 |
| cfa-miR-219-5p | UGAUUGUCCAAACGCAAUUCU | 21 | MIMAT0006611 |
| cfa-miR-23b | AUCACAUUGCCAGGGAUUA | 19 | MIMAT0006612 |
| cfa-miR-27b | UUCACAGUGGCUAAGUUCUGC | 21 | MIMAT0006613 |
| cfa-miR-24 | UGGCUCAGUUCAGCAGGAACAGG | 23 | MIMAT0006614 |
| cfa-miR-151 | UCGAGGAGCUCACAGUCUAGU | 21 | MIMAT0006615 |
| cfa-miR-30d | UGUAAACAUCCCCGACUGGAAGCU | 24 | MIMAT0006616 |
| cfa-miR-30b | UGUAAACAUCCUACACUCAGCU | 22 | MIMAT0006617 |
| cfa-miR-1836 | UAGGCCAUGGUAGAUAGAGAUGG | 23 | MIMAT0006618 |
| cfa-miR-122 | UGGAGUGUGACAAUGGUGUUUG | 22 | MIMAT0006619 |
| cfa-miR-196b | UAGGUAGUUUCCUGUUGUUGGGA | 23 | MIMAT0006620 |
| cfa-miR-183 | UAUGGCACUGGUAGAAUUCACU | 22 | MIMAT0006621 |
| cfa-miR-148a | UCAGUGCACUACAGAACUUUGU | 22 | MIMAT0006622 |
| cfa-miR-129 | CUUUUUGCGGUCUGGGCUUGC | 21 | MIMAT0006623 |
| cfa-miR-335 | UCAAGAGCAAUAACGAAAAAUGU | 23 | MIMAT0006624 |
| cfa-miR-29b | UAGCACCAUUUGAAAUCAGUGUU | 23 | MIMAT0006625 |
| cfa-miR-29a | UAGCACCAUCUGAAAUCGGUUA | 22 | MIMAT0006626 |
| cfa-miR-30e | CUUUCAGUCGGAUGUUUACAGC | 22 | MIMAT0006627 |
| cfa-miR-135a-5p | UAUGGCUUUUUAUUCCUAUGUGA | 23 | MIMAT0010196 |
| cfa-miR-135a-3p | UGUAGGGAUGGAAGCCAUGAAA | 22 | MIMAT0006628 |
| cfa-miR-383 | AGAUCAGAAGGUGAUUGUGGCU | 22 | MIMAT0006629 |
| cfa-miR-1837 | UCUCAGAGGGACUGCGACAUCU | 22 | MIMAT0006630 |
| cfa-miR-130a | CAGUGCAAUGUUAAAAGGGCAU | 22 | MIMAT0006631 |
| cfa-miR-192 | CUGACCUAUGAAUUGACAGCC | 21 | MIMAT0006632 |
| cfa-miR-128 | UCACAGUGAACCGGUCUCUUU | 21 | MIMAT0006633 |
| cfa-miR-7 | UGGAAGACUAGUGAUUUUGUUGU | 23 | MIMAT0006634 |
| cfa-miR-181c | AACAUUCAACCUGUCGGUGAGUU | 23 | MIMAT0006635 |
| cfa-miR-181d | AACAUUCAUUGUUGUCGGUGGGU | 23 | MIMAT0006636 |
| cfa-let-7g | UGAGGUAGUAGUUUGUACAGUU | 22 | MIMAT0006637 |
| cfa-miR-191 | CAACGGAAUCCCAAAAGCAGCU | 22 | MIMAT0006638 |
| cfa-miR-425 | AAUGACACGAUCACUCCCGUUGA | 23 | MIMAT0006639 |
| cfa-miR-23a | AUCACAUUGCCAGGGAUUU | 19 | MIMAT0006640 |
| cfa-miR-27a | UUCACAGUGGCUAAGUUCCG | 20 | MIMAT0006641 |
| cfa-miR-199 | ACAGUAGUCUGCACAUUGGUU | 21 | MIMAT0006642 |
| cfa-miR-708 | AAGGAGCUUACAAUCUAGCUGGG | 23 | MIMAT0006643 |
| cfa-miR-1838 | CCACCAGCUGGCGUUCCCUGG | 21 | MIMAT0006644 |
| cfa-miR-139 | UGGAGACGCGGCCCUGUUGGAA | 22 | MIMAT0006645 |
| cfa-miR-138b | AGCUGGUGUUGUGAAUCAUGCCGA | 24 | MIMAT0006646 |
| cfa-miR-15a | UAGCAGCACAUAAUGGUUUGU | 21 | MIMAT0006647 |
| cfa-miR-16 | UAGCAGCACGUAAAUAUUGGCG | 22 | MIMAT0006648 |
| cfa-miR-17 | ACUGCAGUGAAGGCACUUGUAG | 22 | MIMAT0006649 |
| cfa-miR-19a | UGUGCAAAUCUAUGCAAAACUGA | 23 | MIMAT0006650 |
| cfa-miR-20a | UAAAGUGCUUAUAGUGCAGGUAG | 23 | MIMAT0006651 |
| cfa-miR-19b | UGUGCAAAUCCAUGCAAAACUG | 22 | MIMAT0006652 |
| cfa-miR-92a | UAUUGCACUUGUCCCGGCCUGU | 22 | MIMAT0006653 |
| cfa-miR-138a | AGCUGGUGUUGUGAAUCAGGCCG | 23 | MIMAT0006654 |
| cfa-miR-499 | UUAAGACUUGCAGUGAUGUUU | 21 | MIMAT0006655 |
| cfa-miR-1 | UGGAAUGUAAAGAAGUAUGUA | 21 | MIMAT0006656 |
| cfa-miR-124 | UAAGGCACGCGGUGAAUGCCA | 21 | MIMAT0006657 |
| cfa-miR-320 | AAAAGCUGGGUUGAGAGGGCGA | 22 | MIMAT0006658 |
| cfa-miR-130b | CAGUGCAAUGAUGAAAGGGCAU | 22 | MIMAT0006659 |
| cfa-miR-185 | UGGAGAGAAAGGCAGUUCCUGA | 22 | MIMAT0006660 |
| cfa-miR-1306 | CCACCUCCCCUGCAAACGUCC | 21 | MIMAT0006661 |
| cfa-miR-196a | UAGGUAGUUUCAUGUUGUUGGG | 22 | MIMAT0006662 |
| cfa-miR-148b | UCAGUGCAUCACAGAACUUUGU | 22 | MIMAT0006663 |
| cfa-miR-200c | UAAUACUGCCGGGUAAUGAUGGA | 23 | MIMAT0006664 |
| cfa-miR-1307 | ACUCGGCGUGGCGUCGGUCGUG | 22 | MIMAT0006665 |
| cfa-miR-107 | AGCAGCAUUGUACAGGGCUAU | 21 | MIMAT0006666 |
| cfa-miR-146b | UGAGAACUGAAUUCCAUAGGCU | 22 | MIMAT0006667 |
| cfa-miR-99a | AACCCGUAGAUCCGAUCUUGU | 21 | MIMAT0006668 |
| cfa-let-7c | UGAGGUAGUAGGUUGUAUGGUU | 22 | MIMAT0006669 |
| cfa-miR-125b | UCCCUGAGACCCUAACUUGUGA | 22 | MIMAT0006670 |
| cfa-miR-155 | UUAAUGCUAAUCGUGAUAGGGGU | 23 | MIMAT0006671 |
| cfa-miR-218 | UUGUGCUUGAUCUAACCAUGU | 21 | MIMAT0006672 |
| cfa-miR-574 | CACGCUCAUGCACACACCCACA | 22 | MIMAT0006673 |
| cfa-miR-9 | UCUUUGGUUAUCUAGCUGUAUGA | 23 | MIMAT0006674 |
| cfa-miR-28 | CACUAGAUUGUGAGCUCCUGGA | 22 | MIMAT0006675 |
| cfa-miR-15b | UAGCAGCACAUCAUGGUUUA | 20 | MIMAT0006676 |
| cfa-miR-1839 | AAGGUAGAUAGAACAGGUCUUG | 22 | MIMAT0006677 |
| cfa-miR-26b | UUCAAGUAAUUCAGGAUAGGUU | 22 | MIMAT0006678 |
| cfa-miR-1840 | UCACGUGACGGGCCUCGGCG | 20 | MIMAT0006679 |
| cfa-miR-664 | UGGGCUAGGAAAAAUGAUUGGA | 22 | MIMAT0006680 |
| cfa-miR-194 | UGUAACAGCAACUCCAUGUGGA | 22 | MIMAT0006681 |
| cfa-miR-143 | UGAGAUGAAGCACUGUAGCUC | 21 | MIMAT0006682 |
| cfa-miR-378 | ACUGGACUUGGAGUCAGAAGGC | 22 | MIMAT0006683 |
| cfa-miR-146a | UGAGAACUGAAUUCCAUGGGUU | 22 | MIMAT0006684 |
| cfa-miR-1271 | CUUGGCACCUAGUAAGCACU | 20 | MIMAT0006685 |
| cfa-miR-1841 | AGAGGAAAGCUGGACGGCAAGC | 22 | MIMAT0006686 |
| cfa-miR-103 | AGCAGCAUUGUACAGGGCUAUGA | 23 | MIMAT0006687 |
| cfa-miR-328 | CUGGCCCUCUCUGCCCUUCCGU | 22 | MIMAT0006688 |
| cfa-miR-140 | ACCACAGGGUAGAACCACGGA | 21 | MIMAT0006689 |
| cfa-miR-34a | UGGCAGUGUCUUAGCUGGUUGU | 22 | MIMAT0006690 |
| cfa-miR-497 | CAGCAGCACACUGUGGUUUGU | 21 | MIMAT0006691 |
| cfa-miR-195 | UAGCAGCACAGAAAUAUUGGCA | 22 | MIMAT0006692 |
| cfa-miR-34c | AGGCAGUGUAGUUAGCUGAUUGC | 23 | MIMAT0006693 |
| cfa-miR-186 | CAAAGAAUUCUCCUUUUGGGCU | 22 | MIMAT0006694 |
| cfa-miR-106b | UAAAGUGCUGACAGUGCAGAU | 21 | MIMAT0006695 |
| cfa-miR-93 | CAAAGUGCUGUUCGUGCAGGUAG | 23 | MIMAT0006696 |
| cfa-miR-25 | CAUUGCACUUGUCUCGGUCUGA | 22 | MIMAT0006697 |
| cfa-miR-197 | UUCACCACCUUCUCCACCCAGC | 22 | MIMAT0006698 |
| cfa-miR-193b | CGGGGUUUUGAGGGCGAGAUGA | 22 | MIMAT0006699 |
| cfa-miR-590 | UAAUUUUAUGUAUAAGCUAGU | 21 | MIMAT0006700 |
| cfa-miR-1842 | UGGCUCUGCGAGGUCAGCUCA | 21 | MIMAT0006701 |
| cfa-miR-137 | UUAUUGCUUAAGAAUACGCGU | 21 | MIMAT0006702 |
| cfa-miR-92b | UAUUGCACUCGUCCCGGCCUCC | 22 | MIMAT0006703 |
| cfa-miR-350 | UUCACAAAGCCCAUACACUUUU | 22 | MIMAT0006704 |
| cfa-miR-29c | UAGCACCAUUUGAAAUCGGUUA | 22 | MIMAT0006705 |
| cfa-miR-1843 | ACUGGAGGUCUCUGUCUGGCUU | 22 | MIMAT0006706 |
| cfa-miR-181a | AACAUUCAACGCUGUCGGUGAG | 22 | MIMAT0006707 |
| cfa-miR-181b | AACAUUCAUUGCUGUCGGUG | 20 | MIMAT0006708 |
| cfa-miR-342 | UCUCACACAGAAAUCGCACCCGU | 23 | MIMAT0006709 |
| cfa-miR-345 | CCUGAACUAGGGGUCUGGAGG | 21 | MIMAT0006710 |
| cfa-miR-493 | UGAAGGUCUACUGUGUGCCAG | 21 | MIMAT0006711 |
| cfa-miR-433 | AUCAUGAUGGGCUCCUCGGUGU | 22 | MIMAT0006712 |
| cfa-miR-127 | UCGGAUCCGUCUGAGCUUGGCU | 22 | MIMAT0006713 |
| cfa-miR-136 | ACUCCAUUUGUUUUGAUGAUGGA | 23 | MIMAT0006714 |
| cfa-miR-379 | UGGUAGACUAUGGAACGUAGG | 21 | MIMAT0006715 |
| cfa-miR-411 | AUAGUAGACCGUAUAGCGUACG | 22 | MIMAT0006716 |
| cfa-miR-380 | UAUGUAAUAUGGUCCACGUCU | 21 | MIMAT0006717 |
| cfa-miR-323 | CACAUUACACGGUCGACCUCU | 21 | MIMAT0006718 |
| cfa-miR-329a | AGAGGUUUUCUGGGUUUCUGUUU | 23 | MIMAT0006719 |
| cfa-miR-543 | AAACAUUCGCGGUGCACUUCUU | 22 | MIMAT0006720 |
| cfa-miR-495 | AAACAAACAUGGUGCACUUCUU | 22 | MIMAT0006721 |
| cfa-miR-376a | AUCAUAGAGGAAAAUCCACGU | 21 | MIMAT0006722 |
| cfa-miR-487b | AAUCGUACAGGGUCAUCCACUU | 22 | MIMAT0006723 |
| cfa-miR-382 | AAUCAUUCACGGACAACACUUU | 22 | MIMAT0006724 |
| cfa-miR-485 | AGAGGCUGGCCGUGAUGAAUUCG | 23 | MIMAT0006725 |
| cfa-miR-409 | AAUGUUGCUCGUUGAACCCCU | 21 | MIMAT0006726 |
| cfa-miR-369 | AAUAAUACAUGGUUGGUCUUU | 21 | MIMAT0006727 |
| cfa-miR-410 | AAUAUAACACAGAUGGCCUGU | 21 | MIMAT0006728 |
| cfa-miR-219-3p | AGAAUUGUGGCUGGACAUCUGU | 22 | MIMAT0006729 |
| cfa-miR-126 | CAUUAUUACUUUUGGUACGCG | 21 | MIMAT0006730 |
| cfa-miR-212 | ACCUUGGCUCUAGACUGCUUACU | 23 | MIMAT0006731 |
| cfa-miR-132 | UAACAGUCUACAGCCAUGGUCGC | 23 | MIMAT0006732 |
| cfa-miR-22 | AAGCUGCCAGUUGAAGAACUGU | 22 | MIMAT0006733 |
| cfa-miR-144 | UACAGUAUAGAUGAUGUACUAG | 22 | MIMAT0006734 |
| cfa-miR-193a | UGGGUCUUUGCGGGCGAGAUGA | 22 | MIMAT0006735 |
| cfa-miR-142 | CCCAUAAAGUAGAAAGCACUA | 21 | MIMAT0006736 |
| cfa-miR-10a | UACCCUGUAGAUCCGAAUUUGU | 22 | MIMAT0006737 |
| cfa-miR-152 | UCAGUGCAUGACAGAACUUGG | 21 | MIMAT0006738 |
| cfa-miR-338 | UCCAGCAUCAGUGAUUUUGUUGA | 23 | MIMAT0006739 |
| cfa-miR-1844 | AGGACUACGGACGGGCUGAG | 20 | MIMAT0006740 |
| cfa-miR-21 | UAGCUUAUCAGACUGAUGUUGA | 22 | MIMAT0006741 |
| cfa-miR-423a | UGAGGGGCAGAGAGCGAGACUUU | 23 | MIMAT0006742 |
| cfa-miR-652 | AAUGGCGCCACUAGGGUUGUGC | 22 | MIMAT0006743 |
| cfa-miR-224 | CAAGUCACUAGUGGUUCCGUUU | 22 | MIMAT0006744 |
| cfa-miR-424 | CAAAACGUGAGGCGCUGCUAU | 21 | MIMAT0006745 |
| cfa-miR-503 | UAGCAGCGGGAACAGUACUG | 20 | MIMAT0006746 |
| cfa-miR-542 | UGUGACAGAUUGAUAACUGAAA | 22 | MIMAT0006747 |
| cfa-miR-450b | UUUUGCAAUAUGUUCCUGAAU | 21 | MIMAT0006748 |
| cfa-miR-106a | AAAGUGCUUACAGUGCAGGUAG | 22 | MIMAT0006749 |
| cfa-miR-363 | AAUUGCACGGUAUCCAUCUGUAA | 23 | MIMAT0006750 |
| cfa-miR-361 | UUAUCAGAAUCUCCAGGGGUAC | 22 | MIMAT0006751 |
| cfa-miR-384 | AUUCCUAGAAAUUGUUCACAAU | 22 | MIMAT0006752 |
| cfa-miR-374a | UUAUAAUACAACCUGAUAAGU | 21 | MIMAT0006753 |
| cfa-miR-374b | AUAUAAUACAACCUGCUAAGUG | 22 | MIMAT0006754 |
| cfa-miR-421 | AUCAACAGACAUUAAUUGGGCG | 22 | MIMAT0006755 |
| cfa-miR-98 | UGAGGUAGUAAGUUGUAUUGUU | 22 | MIMAT0006756 |
| cfa-miR-221 | AGCUACAUUGUCUGCUGGGUUU | 22 | MIMAT0006757 |
| cfa-miR-532 | CAUGCCUUGAGUGUAGGACCGU | 22 | MIMAT0006758 |
| cfa-miR-500 | AUGCACCUGGGCAAGGAUUCU | 21 | MIMAT0006759 |
| cfa-miR-660 | UACCCAUUGCAUAUCGGAGUUG | 22 | MIMAT0006760 |
| cfa-miR-502 | AAUGCACCUGGGCAAGGAUUCA | 22 | MIMAT0006761 |
| cfa-miR-676 | CUCUUCAAUCUCAGGACUCGC | 21 | MIMAT0006762 |
| cfa-let-7j | UGAGGUAGUAGAGUGCAGUAGUU | 23 | MIMAT0006763 |
| cfa-miR-20b | CAAAGUGCUCACAGUGCAGGUA | 22 | MIMAT0009830 |
| cfa-miR-18b | UAAGGUGCAUCUAGUGCAGUUA | 22 | MIMAT0009831 |
| cfa-miR-18a | UAAGGUGCAUCUAGUGCAGAUA | 22 | MIMAT0009832 |
| cfa-miR-133c | UUGGUCCCCUUCAACCAGCUG | 21 | MIMAT0009833 |
| cfa-miR-133a | UUGGUCCCCUUCAACCAGCUGU | 22 | MIMAT0009834 |
| cfa-miR-133b | UUUGGUCCCCUUCAACCAGCUA | 22 | MIMAT0009835 |
| cfa-let-7b | UGAGGUAGUAGGUUGUGUGGUU | 22 | MIMAT0009836 |
| cfa-miR-10b | CCCUGUAGAACCGAAUUUGUGU | 22 | MIMAT0009837 |
| cfa-miR-34b | AGGCAGUGUAAUUAGCUGAUUG | 22 | MIMAT0009838 |
| cfa-miR-135b | UAUGGCUUUUCAUUCCUAUGUGA | 23 | MIMAT0009839 |
| cfa-miR-153 | UUGCAUAGUCACAAAAGUGA | 20 | MIMAT0009840 |
| cfa-miR-182 | UUUGGCAAUGGUAGAACUCACACU | 24 | MIMAT0009841 |
| cfa-miR-184 | UGGACGGAGAACUGAUAAGGGU | 22 | MIMAT0009842 |
| cfa-miR-187 | UCGUGUCUUGUGUUGCAGCCGG | 22 | MIMAT0009843 |
| cfa-miR-202 | UUCCUAUGCAUAUACUUCUUUG | 22 | MIMAT0009844 |
| cfa-miR-205 | UCCUUCAUUCCACCGGAGUCUG | 22 | MIMAT0009845 |
| cfa-miR-210 | ACUGUGCGUGUGACAGCGGCUGA | 23 | MIMAT0009846 |
| cfa-miR-214 | ACAGCAGGCACAGACAGGCAGU | 22 | MIMAT0009847 |
| cfa-miR-215 | AUGACCUACGAAUUGAUAGACA | 22 | MIMAT0009848 |
| cfa-miR-216a | UAAUCUCAGCUGGCAACUGUG | 21 | MIMAT0009849 |
| cfa-miR-217 | UACUGCAUCAGGAACUGAUUGGAU | 24 | MIMAT0009850 |
| cfa-miR-222 | AGCUACAUCUGGCUACUGGGU | 21 | MIMAT0009851 |
| cfa-miR-223 | UGUCAGUUUGUCAAAUACCCC | 21 | MIMAT0009852 |
| cfa-miR-301a | CAGUGCAAUAGUAUUGUCAAAGC | 23 | MIMAT0009853 |
| cfa-miR-301b | CAGUGCAAUGAUAUUGUCAAAGC | 23 | MIMAT0009854 |
| cfa-miR-302a | ACUUAAACGUGGAUGUACUUGCU | 23 | MIMAT0009855 |
| cfa-miR-302b | ACUUUAACAUGGAAGUACUUUC | 22 | MIMAT0009856 |
| cfa-miR-302c | UUUAACAUGGGGGUACCUGCUG | 22 | MIMAT0009857 |
| cfa-miR-302d | ACUUUAACAUGGAGGCACUUGC | 22 | MIMAT0009858 |
| cfa-miR-367 | ACUGUUGCUAAUAUGCAACUCU | 22 | MIMAT0009859 |
| cfa-miR-489 | GUGACAUCACAUAUACGGCGGC | 22 | MIMAT0009860 |
| cfa-miR-96 | UUUGGCACUAGCACAUUUUUGCU | 23 | MIMAT0009861 |
| cfa-miR-33b | GUGCAUUGCUGUUGCAUUGC | 20 | MIMAT0009862 |
| cfa-miR-145 | GUCCAGUUUUCCCAGGAAUCCCU | 23 | MIMAT0009863 |
| cfa-miR-200b | CAUCUUACUGGGCAGCAUUGGA | 22 | MIMAT0009864 |
| cfa-miR-200a | CAUCUUACCGGACAGUGCUGGA | 22 | MIMAT0009865 |
| cfa-miR-203 | GUGAAAUGUUUAGGACCACUAG | 22 | MIMAT0009866 |
| cfa-miR-208a | AUAAGACGAGCAAAAAGCUUGU | 22 | MIMAT0009868 |
| cfa-miR-208b | AUAAGACGAACAAAAGGUUUGU | 22 | MIMAT0009869 |
| cfa-miR-451 | AAACCGUUACCAUUACUGAGUU | 22 | MIMAT0009870 |
| cfa-miR-375 | UUUGUUCGUUCGGCUCGCGUGA | 22 | MIMAT0009871 |
| cfa-miR-190a | UGAUAUGUUUGAUAUAUUAGGU | 22 | MIMAT0009872 |
| cfa-miR-190b | UGAUAUGUUUGAUAUUGGGUU | 21 | MIMAT0009873 |
| cfa-miR-147 | GUGUGCGGAAAUGCUUCUGCUA | 22 | MIMAT0009874 |
| cfa-miR-490 | CAACCUGGAGGACUCCAUGCUG | 22 | MIMAT0009875 |
| cfa-miR-211 | UUCCCUUUGUCAUCCUUUGCCU | 22 | MIMAT0009867 |
| cfa-miR-141 | AACACUGUCUGGUAAAGAUGG | 21 | MIMAT0009876 |
| cfa-miR-514 | UGAUUGACACCUCUGUGAGUGGA | 23 | MIMAT0009877 |
| cfa-miR-95 | UUCAACGGGUAUUUAUUGAGCA | 22 | MIMAT0009878 |
| cfa-miR-105a | UCAAAUGCUCAGACUCCUGU | 20 | MIMAT0009879 |
| cfa-miR-105b | UCAAAUGCUCAGACUCCUUG | 20 | MIMAT0010198 |
| cfa-miR-188 | CAUCCCUUGCAUGGUGGAGGGU | 22 | MIMAT0009880 |
| cfa-miR-134 | UGUGACUGGUUGACCAGAGGGG | 22 | MIMAT0009883 |
| cfa-miR-149 | UCUGGCUCCGUGUCUUCACUCCC | 23 | MIMAT0009884 |
| cfa-miR-299 | UGGUUUACCGUCCCACAUACAU | 22 | MIMAT0009885 |
| cfa-miR-362 | AAUCCUUGGAACCUAGGUGUGAGU | 24 | MIMAT0009886 |
| cfa-miR-376b | AUCAUAGAGGAAAAUCCAUGUU | 22 | MIMAT0009887 |
| cfa-miR-376c | GUGGAUAUUCCUUCUAUGUUUA | 22 | MIMAT0009888 |
| cfa-miR-370 | GCCUGCUGGGGUGGAACCUGGU | 22 | MIMAT0009889 |
| cfa-miR-377 | AGAGGUUGCCCUUGGUGAAUUC | 22 | MIMAT0009890 |
| cfa-miR-381 | UAUACAAGGGCAAGCUCUCUGU | 22 | MIMAT0009891 |
| cfa-miR-340 | UUAUAAAGCAAUGAGACUGAUU | 22 | MIMAT0009892 |
| cfa-miR-330 | UCUCUGGGCCUGUGUCUUAGGC | 22 | MIMAT0009893 |
| cfa-miR-326 | CCUCUGGGCCCUUCCUCCAG | 20 | MIMAT0009894 |
| cfa-miR-331 | GCCCCUGGGCCUAUCCUAGAA | 21 | MIMAT0009895 |
| cfa-miR-324 | CGCAUCCCCUAGGGCAUUGGUGU | 23 | MIMAT0009896 |
| cfa-miR-325 | CCUAGUAGGUGUUCAGUAAGUGU | 23 | MIMAT0009897 |
| cfa-miR-346 | UGUCUGCCCGCAUGCCUGCCUCU | 23 | MIMAT0009898 |
| cfa-miR-329b | AACACACCUGGUUAACCUCUUU | 22 | MIMAT0009899 |
| cfa-miR-452 | AACUGUUUGCAGAGGAAACUGA | 22 | MIMAT0009900 |
| cfa-miR-483 | UCACUCCUCCCCUCCCGUCUU | 21 | MIMAT0009901 |
| cfa-miR-487a | AAUCAUACAGGGACAUCCAGU | 21 | MIMAT0009902 |
| cfa-miR-488 | CCCAGAUAAUGGCACUCUCAA | 21 | MIMAT0009903 |
| cfa-miR-432 | UCUUGGAGUAGGUCAUUGGGUGG | 23 | MIMAT0009904 |
| cfa-miR-494 | UGAAACAUACACGGGAAACCUC | 22 | MIMAT0009905 |
| cfa-miR-496 | UGAGUAUUACAUGGCCAAUCUC | 22 | MIMAT0009906 |
| cfa-miR-504 | AGACCCUGGUCUGCACUCUAUC | 22 | MIMAT0009907 |
| cfa-miR-505 | GGGAGCCAGGAAGUAUUGAUGU | 22 | MIMAT0009908 |
| cfa-miR-539 | GGAGAAAUUAUCCUUGGUGUGU | 22 | MIMAT0009909 |
| cfa-miR-544 | AUUCUGCAUUUUUAGCAAGUUC | 22 | MIMAT0009910 |
| cfa-miR-545 | UCAGUAAAUGUUUAUUGGAUG | 21 | MIMAT0009911 |
| cfa-miR-551a | GCGACCCACUCUUGGUUUCCA | 21 | MIMAT0009912 |
| cfa-miR-551b | GCGACCCAUACUUGGUUUCAG | 21 | MIMAT0009913 |
| cfa-miR-568 | AUGUAUAAAUGUAUACACAC | 20 | MIMAT0009914 |
| cfa-miR-578 | CUUCUUGUGCUCUAGAUUGGU | 21 | MIMAT0009915 |
| cfa-miR-582 | UACAGUUGUUCAACCAGUUACU | 22 | MIMAT0009916 |
| cfa-miR-589 | UGAGAACCAUGUCUGAUCAGAG | 22 | MIMAT0009917 |
| cfa-miR-592 | AUUGUGUCAAUAUGCGAUGAUGU | 23 | MIMAT0009918 |
| cfa-miR-599 | GUUGUGUCAGUUUAUCAAAC | 20 | MIMAT0009919 |
| cfa-miR-615 | GGGGGUCCCCGGAGCUCGG | 19 | MIMAT0009920 |
| cfa-miR-628 | AUGCUGACAUAUUUACUAGAGG | 22 | MIMAT0009921 |
| cfa-miR-631 | GACCUGGCCCAGACCUCAGC | 20 | MIMAT0009922 |
| cfa-miR-632 | GUGUCUGUUUCCUGUGGGA | 19 | MIMAT0009923 |
| cfa-miR-653 | GUGUUGAAACAAUCUCUAUUG | 21 | MIMAT0009924 |
| cfa-miR-758 | UUUGUGACCUGGUCCACUAACC | 22 | MIMAT0009925 |
| cfa-miR-671 | UCCGGUUCUCAGGGCUCCACC | 21 | MIMAT0009926 |
| cfa-miR-454 | UAGUGCAAUAUUGCUUAUAGGG | 22 | MIMAT0009927 |
| cfa-miR-802 | CAGUAACAAAGAUUCAUCCUUGU | 23 | MIMAT0009928 |
| cfa-miR-300 | UUGAAGAGAGGUUAUCCUUCGU | 22 | MIMAT0009929 |
| cfa-miR-874 | CUGCCCUGGCCCGAGGGACCGA | 22 | MIMAT0009930 |
| cfa-miR-875 | UAUACCUCAGUUUUAUCAGGUG | 22 | MIMAT0009931 |
| cfa-miR-876 | UGGAUUUCUUUGUGAAUCACCA | 22 | MIMAT0009932 |
| cfa-miR-885 | UCCAUUACACUACCCUGCCUCU | 22 | MIMAT0009933 |
| cfa-miR-665 | ACCAGGAGGCUAAGGCCCCU | 20 | MIMAT0009934 |
| cfa-miR-207 | GCUUCUCCGGUCUCUCCUCCUUC | 23 | MIMAT0009935 |
| cfa-miR-761 | GCAGCAGGGUGAAACUGACACA | 22 | MIMAT0009936 |
| cfa-miR-764 | GGUGCUCACUUGUCCUCCU | 19 | MIMAT0009937 |
| cfa-miR-759 | GCAGAGUGCAAACAAUUUUGCC | 22 | MIMAT0009938 |
| cfa-miR-718 | CUUCCGCCCCGCCGGGCGCCG | 21 | MIMAT0009939 |
| cfa-miR-872 | AAGGUUAUGUGUUAGCUCAAG | 21 | MIMAT0009940 |
| cfa-miR-1199-5p | CCUGAGCCCAGGCCGCGCAG | 20 | MIMAT0031123 |
| cfa-miR-1199-3p | UGCGGCCGGUGCUCAGCCUGC | 21 | MIMAT0031124 |

**Supplementary Table 2.** Differentially expressed miRNAs in dogs with heart diseases compared with healthy dogs

| miRNA | Target protein |  | Healthy |  | MMVD | | | |  | PS | | | |
| --- | --- | --- | --- | --- | --- | --- | --- | --- | --- | --- | --- | --- | --- |
|  |  |  | raw value |  | raw value | FC | p | Regulation |  | raw value | FC | p | Regulation |
| cfa-miR-130b | CYLD (40) |  | 1.52 |  | 2.61 | 2.13^a^ | 0.014 | ↑ |  | 1.07 | 1.36 | 0.297 | ↓ |
| cfa-miR-346 | Bax (54) |  | 0.94 |  | 1.29 | 1.27 | 0.337 | ↑ |  | 2.39 | 2.74^a^ | 0.032 | ↑ |
| cfa-let-7b | Cyclin D2 (33) |  | 7.90 |  | 4.39 | 11.42^a^ | 0.049 | ↓ |  | 5.35 | 5.88^a^ | 0.01 | ↓ |
| cfa-miR-30c | CTGF (31) |  | 2.76 |  | 0.97 | 3.45^a^ | 0.013 | ↓ |  | 1.04 | 3.31^a^ | 0.014 | ↓ |
| cfa-miR-30d | MAP4K4 (45) |  | 2.47 |  | 0.87 | 3.02^a^ | 0.047 | ↓ |  | 1.75 | 1.64 | 0.337 | ↓ |
| cfa-miR-19b | PTEN (47) |  | 3.30 |  | 1.72 | 3.01^a^ | 0.008 | ↓ |  | 2.50 | 1.75 | 0.141 | ↓ |
| cfa-miR-425 | TGFβ1 (44) |  | 3.47 |  | 2.11 | 2.56^a^ | 0.045 | ↓ |  | 2.22 | 2.38 | 0.051 | ↓ |
| cfa-let-7g | THBS1 (48) |  | 2.26 |  | 0.93 | 2.53^a^ | 0.015 | ↓ |  | 1.31 | 1.94 | 0.071 | ↓ |
| cfa-miR-151 | PLM (46) |  | 1.97 |  | 1.05 | 1.89^a^ | 0.023 | ↓ |  | 1.75 | 1.17 | 0.732 | ↓ |
| cfa-miR-375 | PDK-1 (42) |  | 1.23 |  | 0.63 | 1.51^a^ | 0.014 | ↓ |  | 0.87 | 1.28 | 0.126 | ↓ |
| cfa-miR-505 | FGF18 (58) |  | 1.63 |  | 1.35 | 1.21 | 0.46 | ↓ |  | 0.99 | 1.56^a^ | 0.016 | ↓ |

FC, fold change; MMVD, myxomatous mitral valve degeneration; PS, pulmonic stenosis.

MicroRNA target proteins previously reported in other species were shown.

The raw expression levels were shown as log2-transformed value.

↑, up-regulated; ↓, down-regulated.

^a^p < 0.05 compared with healthy group.
